# The burden of psoriasis: a call for awareness

**DOI:** 10.1016/j.eclinm.2021.101114

**Published:** 2021-08-26

**Authors:** EClinicalMedicine

Every August the USA National Psoriasis Foundation (NPS), launches the National Psoriasis Month to spread the knowledge about the etiopathology of psoriasis, to inform about available treatments, and to support and unite those affected. As psoriasis is often mistakenly perceived as infectious and patients can be victims of social stigmatisation, it is pivotal to promote awareness of what psoriasis is and is not and to fight the discrimination suffered by people living with it.

Psoriasis is a chronic, immune-mediated inflammatory skin disease possibly resulting from a defect in proliferation and differentiation of skin cells associated with inflammatory cell infiltration. This process causes skin cells to accumulate and form plaques that itch, burn, and sting. These plaques appear on any part of the body, but are most often found on the elbows, knees, and scalp. Psoriasis scaly patches can range from a few spots of dandruff-like scaling to major eruptions that cover large areas, including the entire body. In addition, according to the NPS there are at least five types of psoriasis. Including the most common form with plaques, psoriasis also occurs in guttate, inverse, pustular, and erythrodermic forms. Of note, psoriasis is not limited to the skin, but can produce systemic effects and can be associated with psoriatic arthritis, cardiovascular disorders, and diabetes, as reported by WHO in the Global Report on Psoriasis, in 2016.

According to the US Centers for Disease Control and Prevention, psoriasis is one of the most common chronic cutaneous dermatitis, which, in 2021, affects more than 7•5 million people in the USA and approximately 125 million people worldwide. Given the poor characterisation of this disease, those with psoriasis also suffered from a lack of effective treatments. From the beginning of the 20th century, different manifestations of psoriasis were treated with topical corticosteroids, phototherapy, systemic and topical retinoids, and narrowband UVB. In the early 2000s, the serendipitous discovery of the benefit of cyclosporine led to the development of drugs first targeting T-cells and subsequently to the cytokine mediators. Targeting cytokines, paved the way to the development, in 2021, of the drug bimekizumab. Research from the University of Manchester, UK, and Salford Royal NHS Foundation Trust, UK, published this July in *The New England Journal of Medicine*, report that a monthly injection of bimekizumab, can reduce psoriasis plaques of 90% after only 16 weeks of treatment. Bimekizumab*,* compared with other biologics like secukinumab and adalimumab, stands out with the highest rate of skin clearance and the ability to treat psoriatic arthritis, one of the most common and painful comorbidities of psoriasis. As the cause of psoriasis is still unknown, treatments are only available to control symptoms and often need to be taken continuously.

Jankowiak and colleagues, from Medical University of Białystok Poland, in a study published in *Dermatology* in August, 2020, highlight how psoriasis can also significantly affect quality of life of the patients, causing great physical, emotional, and social burden. Disfiguring manifestations of psoriasis can lead to depression and other psychological problems. The presence of severe psoriatic plaques that eventually can crack and bleed and that are highly visible on the body, might trigger a negative reaction in others and, since psoriasis is wrongly considered infectious by many, some patients might be stigmatised by people in their communities. Patients with psoriasis can also experience self-stigmatisation, and thus start to avoid others, limit their social relations, abandon their work, and ultimately, completely alienate themselves from the society. Moreover, not only psoriatic lesions visible to others, but also those that are usually covered and unseen (eg, in the genital area) can be a cause of shame and isolation.

Improving awareness of psoriasis among the lay public will help to finally debunk the myth of psoriasis as contagious. An informed understanding of this chronic skin disease would broaden social acceptance and provide better support for people living with this condition. National Psoriasis Month, this August, is an opportunity to promote awareness of the physical, emotional, and social challenges that people with this chronic disease are still facing. Health care in addition to the management of the skin lesions and screening for associated comorbidities should also require psychological evaluation and support for patients with psoriasis. Let us eradicate once and for all the myth of psoriasis as an infectious disease.

*EClinicalMedicine*

